# Supplementary material for: Blood concentrations of mediators released from activated neutrophils are related to the severity of alcohol-induced liver damage
Source: PLoS One. 2023 Jan 6;18(1):e0280068. doi: 10.1371/journal.pone.0280068 (PMC9821433; doi:10.1371/journal.pone.0280068)
Supplement: Attachment — Submitted filename: Kasztelan-Szczerbinska_our rebuttal letter.docx [file pone.0280068.s001.docx]

Lublin, December 4th, 2022

**Our rebuttal letter,**

**Manuscript PONE-D-22-28565, entitled:** *Blood concentrations of mediators released from activated neutrophils are related to the severity of alcohol-induced liver damage*

We would like to thank both Reviewers for allowing us to improve our paper and submit a revised draft of our manuscript titled: *Blood concentrations of mediators released from activated neutrophils are related to the severity of alcohol-induced liver damage,* to Plos One. We appreciate the time and effort that the reviewers have dedicated to providing their valuable feedback and comments on our manuscript. We have incorporated changes reflecting all the suggestions advisable by the reviewers. The changes have been highlighted in yellow within the manuscript.

**Our response to Reviewer #1: (in blue)**

**Comment from Reviewer #1**

„Abstract
It is not usual to use the name "conventional" markers of inflammation. Please replace with a more adequate term.”

Our response: We change to **routine** markers instead of using conventional markers, although the term conventional is widely used by researchers, for example:

1. Jiang et al. Novel Surrogate Markers of CNS Inflammation in CSF in the Diagnosis of Autoimmune Encephalitis. Front. Neurol. 2020,10:1390. doi: 10.3389/fneur.2019.01390 „Supportive findings indicating AE include the following **conventional** surrogate markers of neuroinflammation on CSF: oligoclonal bands, raised protein level, and monocytosis”;
2. Guz G, Colak B, Hizel K, et al. Procalcitonin and **Conventional Markers** of Inflammation in Peritoneal Dialysis Patients and Peritonitis. *Peritoneal Dialysis International*. 2006;26(2):240-248. doi:[10.1177/089686080602600221](https://doi.org/10.1177/089686080602600221)

**Comment from Reviewer #1** „NE was related to MELD and mDF scores. What do you mean by this?”
Our response: Thank you for pointing this out. The sentence was corrected as follows- NE correlated with MELD and mDF scores.

**Comment from Reviewer #1** „Keywords? Blood biomarkers. Broad term, should be replaced

Our response: We have replaced the term with neutrophil-derived biomarkers.

**Comment from Reviewer #1** „Introduction Very clear and concise. However, this is the first time I've encountered a goal like this. He is really clear, however, the questions asked are not usual for the original article. If this is the policy of the journal, then it is ok. If not, it is necessary to modify the questions in, for example, aim: 2.

Our response: Yes, we tried to be clear and concise, therefore we presented all the details of our research in the simplest possible way starting from questions that we were going to answer by conducting our study.

**Comment from Reviewer #1** „Ln 86-87. Conducted study... This should be transferred to Material and methods”
Our response: The sentence has been transferred to Material and methods, as recommended by the Reviewer.

**Comment from Reviewer #1** „Material and methods
Ln 139 Please check the names of the parameters and add the correct abbreviations or description of the abbreviations where they are missing. For example: alanine aminotransferase is not A1AT, but ALT. Guanosine-5'-triphosphate is GTP. In your case it is gamma-glutamyl transpeptidase. Do you mean transaminotrasferase (GGT)?”

Our response: We apologize for the mistakes. All of them have been corrected as follows: AST/ALT ratio; alanine aminotransferase – ALT; gamma-glutamyl transpeptidase- GGT.

**Comment from Reviewer #1** „Why did you mention alkaline phosphatase in the assessment of liver function, when it is an organospecific enzyme for bones?”

Our response: While we appreciate the Reviewer’s feedback, we respectfully disagree. The two main blood sources of alkaline phosphatase (ALP) include the liver and bones. High levels of ALP may indicate bile duct disorders and should be checked together with GGT to confirm the presence of cholestasis. As we and other researchers have confirmed in previous studies, a cholestatic pattern with elevated ALP levels and intrahepatic cholestasis is a poor prognostic indicator in patients with ALD (Kasztelan-Szczerbinska et al. Alkaline phosphatase: the next independent predictor of the poor 90-day outcome in alcoholic hepatitis. Biomed Res Int. 2013;2013:614081. doi: 10.1155/2013/614081. Epub 2013 Sep 17. PMID: 24151614; PMCID: PMC3789301; Tung BY, Carithers RL Jr. Cholestasis and alcoholic liver disease. Clin Liver Dis. 1999 Aug;3(3):585-601. doi: 10.1016/s1089-3261(05)70086-6. PMID: 11291240; Spahr et al. Early liver biopsy, intraparenchymal cholestasis, and prognosis in patients with alcoholic steatohepatitis. BMC Gastroenterol. 2011 Oct 28;11:115. doi: 10.1186/1471-230X-11-115. PMID: 22035247; PMCID: PMC3228746; Axley et al. Severe Alcoholic Hepatitis: Atypical Presentation with Markedly Elevated Alkaline Phosphatase. J Clin Transl Hepatol. 2017 Dec 28;5(4):414-415. doi: 10.14218/JCTH.2017.00044. Epub 2017 Sep 3. PMID: 29226108; PMCID: PMC5719199)

**Comment from Reviewer #1** „What is INR?”
Our response: The abbreviation has been added and explained as follows- the international normalized ratio (INR).

**Comment from Reviewer #1** „Ln, 141 it is necessary to equalize the WBC count and RBC count. Abbreviations are missing again.”

Our response: The sentence has been corrected and the abbreviations have been added as follows: red blood cell- RBC count, platelet- PLT count, and white blood cell- WBC count.

**Comment from Reviewer #1** „Ln 145 Replace the word conventional.”

Our response: We have replaced the word with **routine** (markers) instead of using conventional (markers), although the term conventional is widely used by researchers, as we have explained above.

**Comment from Reviewer #1**

**Comment from Reviewer #1** „I believe that the methods should be classified into several groups. Please form adequate titles and subtitles. You can't write together about blood and serum parameters, then there is a division of patients, certain criteria and symptoms….”

Our response: We agree with the Reviewer’s assessment. The methods have been corrected and divided into different groups as follows 2.3.1, 2.3.2, 2.3.3, and 2.3.4. Furthermore, the methods have been separated from the patient description (Material).

**Comment from Reviewer #1** „Table 2. GTP, please correct this cardinal error. Here it is now ALT, and in methods A1AT. What does the mark kom/uL represent? If the international normalized ratio (INR) is the unit for prothrombin time, there cannot be an INR in the table. It's the same for you if you wrote mmol/L instead of the glucose value. Ln instead of 700 x g specify rpm.”

Our response: We apologize for all the mistakes. They have been corrected as follows: GTP into GGT, kom/uL into cells/uL, and 700 x g to 3000 rpm.

**Comment from Reviewer #1**

Table 4. Please enter a numerical value for each p value, and not combine p and significant levels. Place a mark next to the p value and mark the level of significance (p<0.000) under the table.

Our response: Our statistical program which is MedCalc® Statistical Software version 20.113 (MedCalc Software Ltd, Ostend, Belgium; https://www.medcalc.org; 2022) for numerical values as low as P < 0.0001 shows only this kind of statistical result. Below we present the result obtained from the program.

Mann-Whitney test (independent samples)

| Sample 1 | |
| --- | --- |
| Variable | NE |
| Filter | ALD=1 |
| Sample 2 | |
| Variable | NE |
| Filter | ALD=0 |

|  | Sample 1 | Sample 2 |
| --- | --- | --- |
| Sample size | 64 | 24 |
| Lowest value | [69.4154](cmd:SHOWXMINMAX?78) | [46.8437](cmd:SHOWYMINMAX?8) |
| Highest value | [637.5770](cmd:SHOWXMINMAX?65) | [361.0850](cmd:SHOWYMINMAX?68) |
| Median | 255.1155 | 107.2220 |
| 95% CI for the median | 213.1796 to 301.4111 | 94.3845 to 120.2510 |
| Interquartile range | 159.3660 to 380.8010 | 87.3854 to 138.0075 |

| Hodges-Lehmann median difference | -126,8520 |
| --- | --- |
| 95% Confidence interval | -187,2400 to -69,5905 |

## Mann-Whitney test (independent samples)

| Average rank of first group | 52.6406 |
| --- | --- |
| Average rank of second group | 22.7917 |
| Mann-Whitney U | 247.00 |
| Large sample test statistic Z | 4.881 |
| Two-tailed probability | P < 0.0001 |

Sunday, December 4, 2022 15:42
MedCalc^®^ Statistical Software version 20.113 (MedCalc Software Ltd, Ostend, Belgium; https://www.medcalc.org; 2022)

**Comment from Reviewer #1** „Tables 5 and 6 are not described. Remove n from Table 6 to make the table smaller. Please eliminate the green coloring and mark significant differences with *. The description of the images is also not given. It looks like you presented the data as figures next to the table. The figures are not described at all. It is necessary to standardize all tables. It is better to eliminate the green ones, put a sign * and write an explanation for significance under the table. In general, a lot of data in the results is displayed chaotically.

Our response: The Tables and Figures have been corrected according to the Reviewer's suggestions. Table 6-9- n has been removed to make the tables smaller and unify them, the green color has been deleted, statistical significance has been indicated with *, and an explanation for significance has been placed under the tables.

Also, a description of the figures has been included.

**Comment from Reviewer #1** 3.5. There are no data.
Our response: Since no significant differences in the levels of studied neutrophilic biomarkers were observed between patients with or without ALD complications, we just commented on this without showing data. Nevertheless, the data can be presented and they have been included in the revised manuscript text.

**Comment from Reviewer #1** „Discussion
The first page of the discussion (ln 359-385) is a general story. This could have been the Introduction. The discussion is well written.”

Our response: We appreciate the Reviewer’s valuable comments and kind words regarding the discussion.

We would like to thank Reviewer #1 once again for the comprehensive evaluation of our manuscript.

**Our response to Reviewer #2 (in blue)**

**Comment from Reviewer #2** „I have just one recommendation to the authors is :
the type of the study must mention in the abstract”

Our response: We appreciate the time and effort that Reviewer #2 has dedicated to providing valuable feedback on our manuscript.

The type of our study (prospective one) has been mentioned in the abstract.

We would like to thank Reviewer #2 once again for the comprehensive evaluation of our manuscript.

**Our response to Editor’s comments (in blue)**

1. Please ensure that your manuscript meets PLOS ONE's style requirements, including those for file naming. The PLOS ONE style templates can be found at
   <https://journals.plos.org/plosone/s/file?id=wjVg/PLOSOne_formatting_sample_main_body.pdf> and 
   <https://journals.plos.org/plosone/s/file?id=ba62/PLOSOne_formatting_sample_title_authors_affiliations.pdf>.

Our response: We have checked PLOS One’s style requirements and followed them.

2. Your ethics statement should only appear in the Methods section of your manuscript. If your ethics statement is written in any section besides the Methods, please move it to the Methods section and delete it from any other section. Please ensure that your ethics statement is included in your manuscript, as the ethics statement entered into the online submission form will not be published alongside your manuscript.

Our response: The ethics statement has been moved to the Methods section and deleted from the other section.

3. We note you have included a table to which you do not refer in the text of your manuscript. Please ensure that you refer to Table 3 in your text; if accepted, production will need this reference to link the reader to the Table.

Our response: We have referred to Table 3 in the text of our manuscript.

We would like to thank the Editor for the comprehensive evaluation of our manuscript.

Kind regards

On behalf of all authors of the manuscript

**Beata Kasztelan-Szczerbinska, MD, Ph.D.**

Department of Gastroenterology with Endoscopy Unit

Medical University of Lublin, Poland
